# Effect of Elevated Deformation Temperatures on Microstructural and Tensile Behavior of Si-Al Alloyed TRIP-Aided Steel

**DOI:** 10.3390/ma13225284

**Published:** 2020-11-22

**Authors:** Aleksandra Kozłowska, Adam Grajcar

**Affiliations:** Department of Engineering Materials and Biomaterials, Silesian University of Technology, 18a Konarskiego Street, 44-100 Gliwice, Poland; aleksandra.kozlowska@polsl.pl

**Keywords:** transformation-induced plasticity, stability of retained austenite, multiphase steel, plastic deformation

## Abstract

The influence of elevated deformation temperatures on the relationships between the microstructure and mechanical properties in a hot-rolled Si-Al-alloyed transformation-induced plasticity (TRIP)-aided steel was studied in a static tensile test. The morphological features of specimens deformed at the different temperatures were characterized by different microstructural techniques: optical microscopy (OM), scanning electron microscopy (SEM), X-ray diffraction (XRD), and transmission electron microscopy (TEM). An increase in the deformation temperature from 20 to 200 °C resulted in the reduced effectiveness of the TRIP effect, due to the increasing mechanical stability of the γ phase. The gradual transformation of retained austenite into martensite expressed by a progressive increase in the work hardening exponent (*n*) led to a beneficial balance of strength, uniform elongation and total elongation. The best product of UTS × TEl = 17,805 MPa% showed the sample deformed at 20 °C with a peak *n* value amounting to 0.3.

## 1. Introduction

In the last decade, the automotive industry has paid special attention to the weight reduction of automobile structural parts and improvement of passengers’ passive safety. An increased ability to absorb energy and a beneficial strength–ductility balance can be provided by the transformation-induced plasticity (TRIP) effect, which is related to strain-induced martensitic transformation (SIMT) [1,2,3]. It leads to an increased work-hardening rate, delayed necking, and enhanced ductility [4,5,6]. Steels showing the TRIP effect are classified as advanced high strength steels (AHSS). Their microstructure consists of ferrite, bainite, and dispersed retained austenite of different morphologies [7,8,9]. The mechanical stability of the retained austenite is related to its resistance against strain-induced martensitic transformation depending on grain size [10,11], morphology [12,13], type of surrounding phases [14,15], stress state [16,17], strain rate [18,19,20,21], and also on the stacking fault energy (SFE) affected by the deformation temperature [13,22,23,24,25,26]. 

The design of TRIP steels aims to optimize the amount and stability of γ phase. It can be done through the modification of chemical composition and process parameters which provide the desirable microstructure with the tailored stability of retained austenite [6,7]. A part of this metastable phase is subjected to the SIMT during forming operations such as stamping, bending, drawing, etc. The remaining amount of this phase is able to undergo martensitic transformation during crush events. In both cases, some amount of heat is generated in the deformed sheets [27,28]. The stability of retained austenite gradually increases with increasing deformation temperature [23,26,29,30]. Thus, it is very important to assess the effectiveness of the SIMT at elevated deformation temperatures.

Recent studies have mainly concerned the kinetics of SIMT at room deformation temperature [3,4,6,7,8,9,10,13,14,15,16,17]. However, some results concerning the effectiveness of the TRIP effect at reduced and elevated deformation temperatures are available in the literature [22,23,26,27,28,29,30]. Mukherjee et al. [30] investigated the stability of the γ phase in Fe-0.4C-1.48Mn-0.49Si-0.96Al steel deformed at 20 and 150 °C. They noticed a slight decrease in ultimate tensile strength (UTS) for the higher deformation temperature. However, the opposite tendency was observed for the total elongation (TE). It was related to the progressive SIMT at the elevated deformation temperature. Most of the previous studies have concerned the characterization of mechanical properties at selected deformation temperatures. However, less attention was paid to detailed studies of microstructure after deformation at elevated temperatures, which reflect operating conditions of auto body sheets, i.e., 20–200 °C. Results presented in the literature usually have not included a quantitative analysis of the stability of retained austenite at elevated temperatures, especially for Nb-microalloyed TRIP steels.

In the present study, the stability of the γ phase in a multiphase Si-Al-alloyed steel refers to its SIMT as a function of tensile temperature from 20 to 200 °C. The previous studies have examined cold-rolled steel grades at room temperature and at selected reduced or elevated temperatures. The influence of the temperature factor on the mechanical stability of retained austenite and the resulting deformation behavior of a microalloyed hot-rolled TRIP steel has not yet been investigated in detail. The present study provides both qualitative and quantitative analyses of the stability of retained austenite at elevated temperatures. The detailed analysis of this issue is also important from a technological point of view due to the operating conditions of steel sheets.

## 2. Material and Experiments 

A laboratory-produced TRIP-aided steel of the chemistry shown in Table 1 was studied in this work. Silicon was added together with aluminum to avoid a negative effect of the first element on a surface quality of galvanized steel sheets. The grain refinement effect was ensured by small additions of Nb and Ti, which form disperse carbonitrides during hot-working.

The alloy was vacuum induction melted and cast under an Ar atmosphere (Institute for Ferrous Metallurgy, Gliwice, Poland). The ingot was homogenized at 1200 °C, forged in a temperature range 1200–900 °C to a thickness of 22 mm and then hot-rolled in a temperature range 1200–900 °C to a thickness of 4.5 mm. Finally, the plate sample was thermomechanically-rolled in 3 passes (at deformation temperatures: 1050, 950 and 850 °C) to 2 mm flat samples. After that, the samples were controlled cooled to 700 °C and then more slowly to 600 °C within 60 s. Next, accelerated cooling (27 °C/s) to 450 °C was applied, where the sheet samples were held for 600 s. The detailed parameters of the processing route can be found in [31].

**Figure 1:**
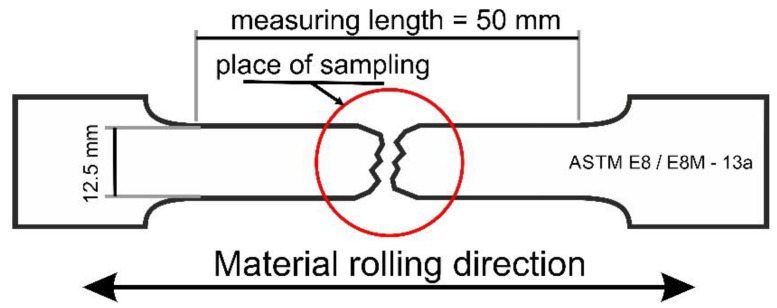
Tensile sample with the marked area of sampling for microstructural analysis.

The microstructures of the steel prior to and after static tensile deformation performed at the following temperatures: 20, 60, 100, 140, and 200 °C were examined by different microstructural techniques (OM, SEM, and TEM). X-ray diffraction patterns were provided to determine the amount of γ phase. Samples were cut from a necking area (Figure 1). Standard metallographic procedures were applied to prepare the samples for microscopic observations [32]. The multiphase microstructure of the investigated samples was revealed using 4% nital (SEM) and sodium pyrosulfate (OM) reagents. A Leica MEFa light microscope (Wetzlar, Germany) was used for the metallographic observations. The SEM observations were carried out by means of a Zeiss Supra 25 microscope (Carl Zeiss AG, Jena, Germany) working in a secondary electron imaging (SE) mode. The detailed observations of microstructural changes were performed using a Titan 80-300, FEI S/TEM microscope operating at an accelerating voltage of 300 kV.

The diffraction studies were performed by Panalytical X’Pert Pro MPD diffractometer (PANalytical, Almelo, The Netherlands) equipped with the PIXcel 3D detector on the diffracted beam axis with Co radiation. The step size of 0.02626° per second in the 2θ range from 40° to 115° was applied. The amount of retained austenite was estimated by comparing the intensities of the (111)γ, (002)γ, (311)γ, (110)α, (002)α, and (211)α peaks. The quantitative determination of the γ phase was realized by using the Averbach–Cohen method [33,34].

The tensile tests were performed using an INSTRON 1195 universal testing machine (INSTRON, Norwood, MA, USA) equipped with a thermal chamber. Standard tensile samples with 12.5 mm width, 2 mm thickness, and 50 mm gauge length [35] were machined from the thermomechanically processed sheet. The tensile sample with the marked area of sampling for microstructural analysis is shown in Figure 1. The tests were performed up to rupture at temperatures ranging from 20 to 200 °C at a strain rate of 10^−3^s^−1^ and a traverse velocity of 3 mm/s. Three specimens were used for each temperature. The true stress–true strain curves were calculated by means of the Equations (1) and (2) providing the σ-ε data in the uniform elongation region:

(1) σ=s(1+e)

(2) ε=ln(1+e)

where σ and ε are true stress and true strain, respectively; and *s* and *e* are nominal stress and strain. The strain hardening exponent (*n*) was calculated using Equation (3):

(3) $$n=\frac{d\left(ln\sigma \right)}{d\left(ln\varepsilon \right)}$$

## 3. Results and Discussion

### 3.1. Microstructural Characterization

**Figure 2:**
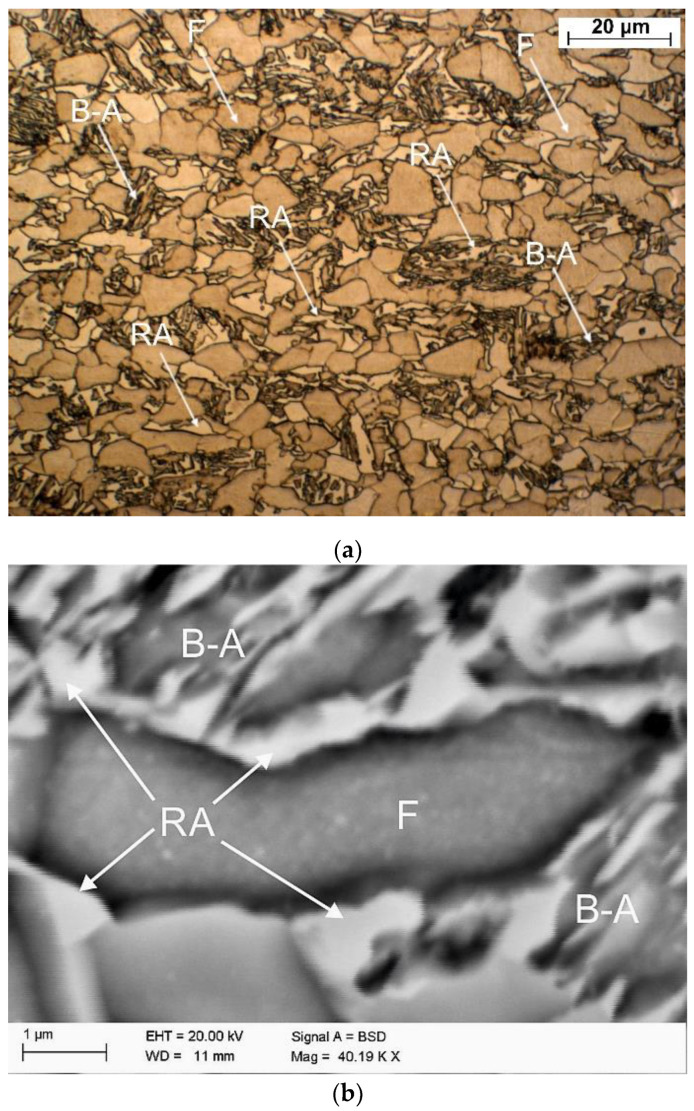
Microstructure of investigated steel in the initial state: (**a**) LM—light microscopy, (**b**) SEM; RA—retained austenite, B–A—bainitic–austenitic areas, F—ferrite.

The initial thermomechanically processed sample is shown in Figure 2. The microstructure contains ferrite surrounded by bainitic–austenitic islands (BA) and retained austenite (RA). The brightest grains and thin layers are represented by retained austenite (Figure 2a). The largest grains are characteristic of ferrite. The darkest lath regions are represented by bainite. The γ phase is homogeneously distributed in the matrix. Large blocky grains of RA are located near ferrite grains, while thin layers of this phase are located inside the bainitic areas. The morphological types of RA can be easily distinguished using higher magnifications provided by SEM (Figure 2b). The amount of γ phase estimated by the XRD method was ca. 15% [36].

**Figure 3:**
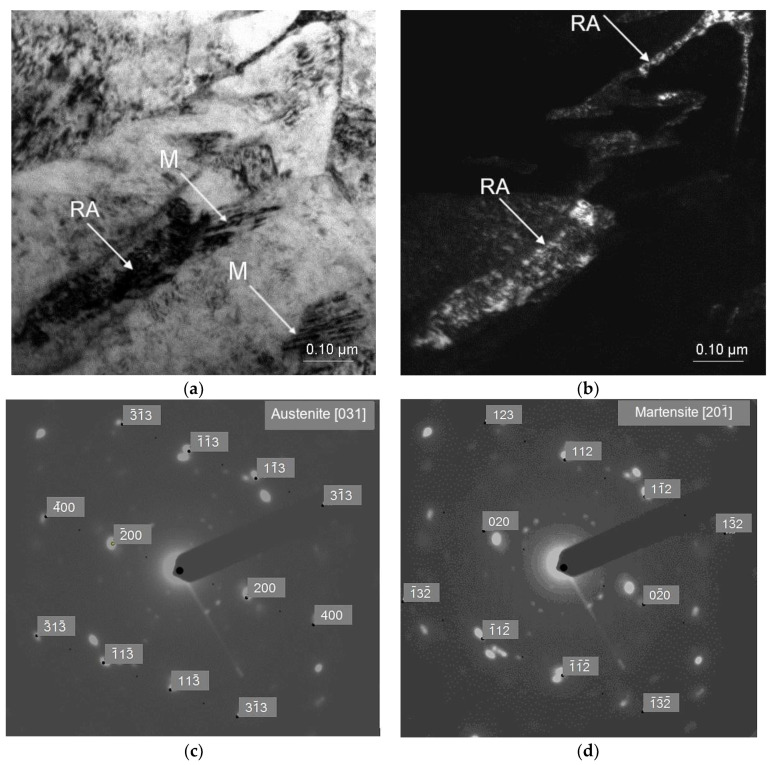
Elongated grain of retained austenite partially transformed into martensite in the specimen deformed at 20 °C: bright field (BF)—(**a**), dark field (DF) of retained austenite (**b**), selected area diffraction pattern of retained austenite (**c**), selected area diffraction pattern of martensite (**d**).

**Figure 4:**
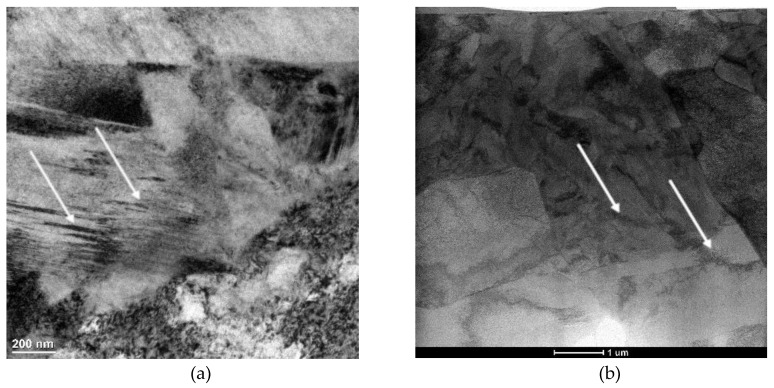
TEM image of the specimen deformed at 200 °C: martensitic laths (bright field)—(**a**), dislocation cells (high-angle annular dark-field imaging (HAADF) scanning-transmission electron mode (STEM))—(**b**).

**Figure 5:**
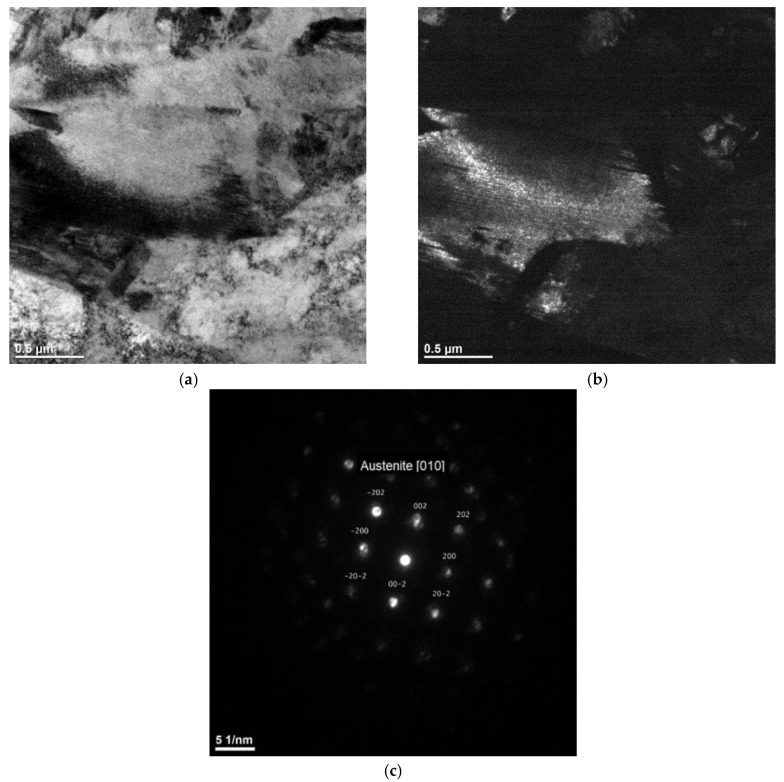
Blocky grain of partially untransformed retained austenite in the specimen deformed at 200 °C: bright field (BF)—(**a**), dark field (DF) of RA (**b**), selected area diffraction pattern of RA (**c**).

The influence of deformation temperature on the morphological details of retained austenite was investigated using a TEM technique. The TEM micrographs of the specimens deformed at 20 and 200 °C are shown in Figure 3, Figure 4 and Figure 5. Transmission electron microscopy technique is very helpful in showing some details of strain-induced martensitic transformation. Figure 3 shows an elongated grain of retained austenite located in the bainitic area, which was partially transformed into martensite as a result of plastic deformation. The outer areas maintained stability whereas the SIMT took place in the central RA part because the grain boundary areas are more enriched in carbon than the central regions [37,38,39]. It can also be seen that the large RA grain was fragmented into a few smaller parts by freshly formed martensite, which contributed to a further increase in its stability (Figure 3b). The grain boundaries act as obstacles to the movement of dislocations generated during the SIMT. Moreover, in small austenite grains, a growth of fresh martensite laths is limited by grain boundaries [37,38]. Chiang et al. [3] also observed that an extent of martensitic transformation induced by strain in 0.17C-1.5Mn-1.53Si steel was related to the morphology, grain size, phase location, and C enrichment in the γ phase. The presence of retained austenite and martensite was confirmed by selected area diffraction (SAED) in Figure 3c,d, respectively. 

The evidence of strain-induced martensitic transformation at 200 °C was confirmed by TEM (Figure 4 and Figure 5). Some martensitic laths can be observed in Figure 4a. Moreover, as a result of the elevated deformation temperature, the presence of dislocation cells in ferrite (Figure 4b) was revealed by the scanning-transmission electron mode (STEM) technique using high-angle annular dark-field imaging (HAADF). Figure 5a,b show the blocky grain of partially untransformed retained austenite. This means that at the highest deformation temperature (200 °C), the strain-induced martensitic transformation only partially takes place depending on local structural conditions, such as the grain size, grain morphology, stress and strain levels, and a chemical composition in an individual microregion. The occurrence of retained austenite was confirmed by SAED (Figure 5c).

**Figure 6:**
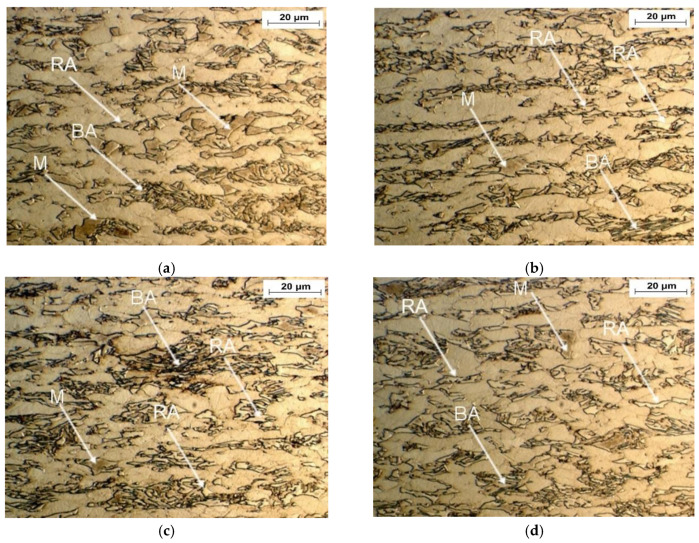
Optical micrographs of specimens deformed at different temperatures: 20 (**a**), 60 (**b**), 100 (**c**), and 200 °C (**d**). RA—retained austenite, BA—bainitic-austenitic islands, M—martensite.

**Figure 7:**
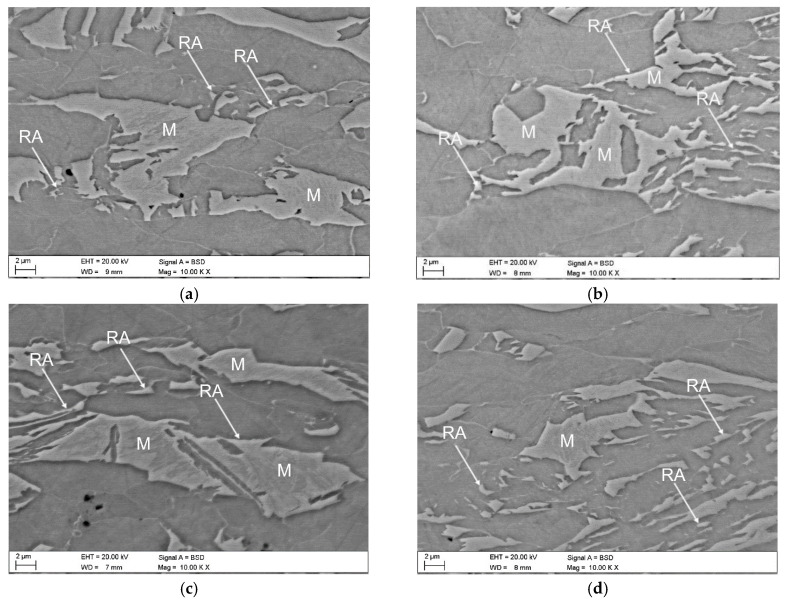
SEM micrographs of specimens deformed at temperatures: 20 (**a**), 60 (**b**), 100 (**c**), and 200 °C (**d**).

Optical and SEM micrographs of the specimens deformed at elevated temperatures ranging from 20 to 200 °C are presented in Figure 6 and Figure 7, respectively. The significant amount of retained austenite transformed into martensite at 20 °C confirmed the high intensity of strain-induced martensitic transformation occurring at this temperature (Figure 6a and Figure 7a). Increasing the deformation temperature above 20 °C resulted in a gradual increase in the stability of retained austenite (Figure 6b,c and Figure 7b,c). This is especially noticeable for the sample deformed at the highest temperature—200 °C (Figure 6d and Figure 7d). An increase in the deformation temperature led to the rise of the stacking fault energy (SFE) of the γ phase [40], which resulted in the lower intensity of SIMT. At the highest deformation temperature, only the largest grains of the RA partially transformed into martensite. Interlath retained austenite remained stable (Figure 6d and Figure 7d).

### 3.2. Retained Austenite Amount

**Figure 8:**
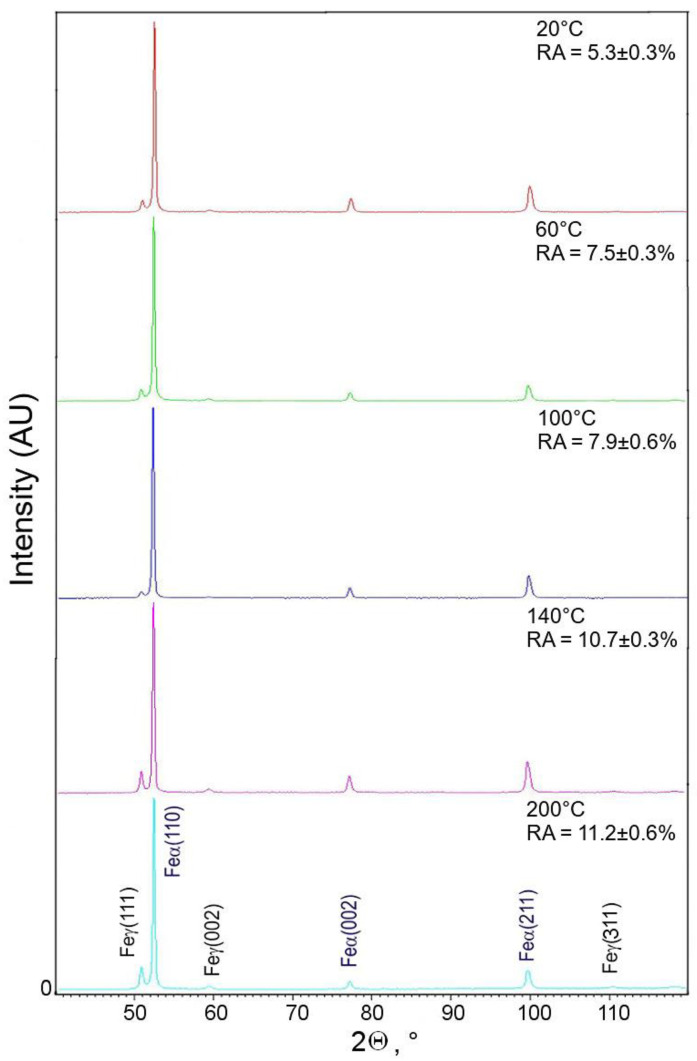
X-ray diffraction patterns of samples deformed at different temperatures.

For the samples deformed at 140 and 200 °C, three body-centered cubic (bcc) diffraction peaks: (110)_α_, (002)_α_, and (211)_α_, and three face-centered cubic (fcc) diffraction peaks: (111)_γ_, (002)_γ_, and (311)_γ_ were detected (Figure 8).

**Figure 9:**
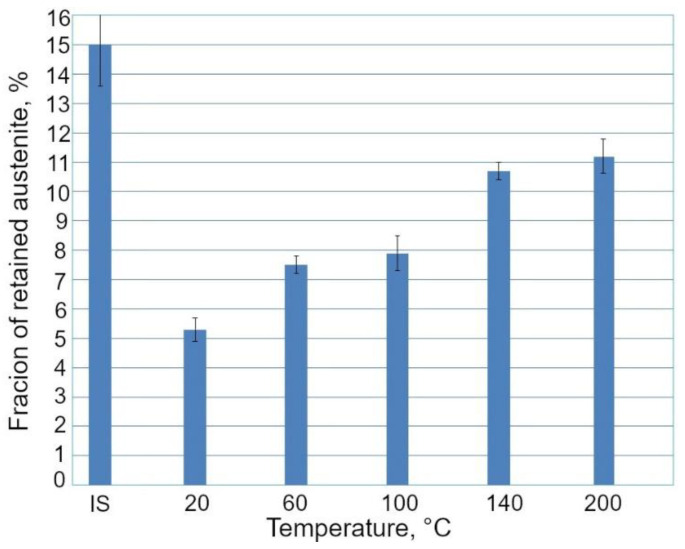
Fraction of retained austenite (RA) in the initial state (IS) and after deformation at different temperatures.

**Figure 10:**
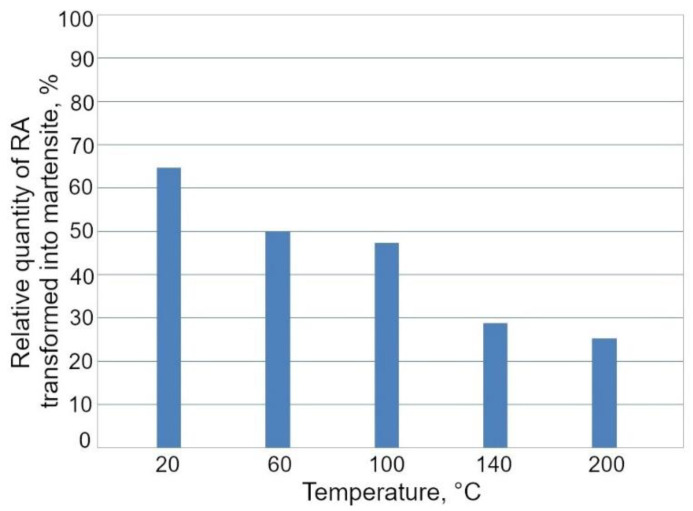
Relative quantity of retained austenite (RA) transformed into martensite at different temperatures.

The fcc diffraction peaks correspond to the retained austenite, while the other ones are generated by the α phases, i.e., ferrite, bainite, and martensite. In the case of the specimens deformed at 20, 60, and 100 °C, only the (111)_γ_ and (002)_γ_ diffraction peaks corresponding to the RA were detected. The SIMT occurrence can be visualized by decreasing the austenite peak intensities. Results of XRD analysis obtained for the investigated steel showed that a volume fraction of γ phase, which transformed into martensite, decreased with increasing deformation temperature (Figure 9). The lowest amount of γ phase maintained stability in the specimens deformed at 20 °C (5.3%), whereas the highest amount of RA (11.2%) was detected after deformation at 200 °C. In the case of the specimen deformed at 20 °C, ca. 65% of the initial amount of RA was transformed, whereas for the specimen deformed at 200 °C, only ca. 25% of RA transformed into martensite (Figure 10). Hence, the clear dependance between the deformation temperature and mechanical stability of the γ phase was evidenced.

### 3.3. Mechanical Properties

**Figure 11:**
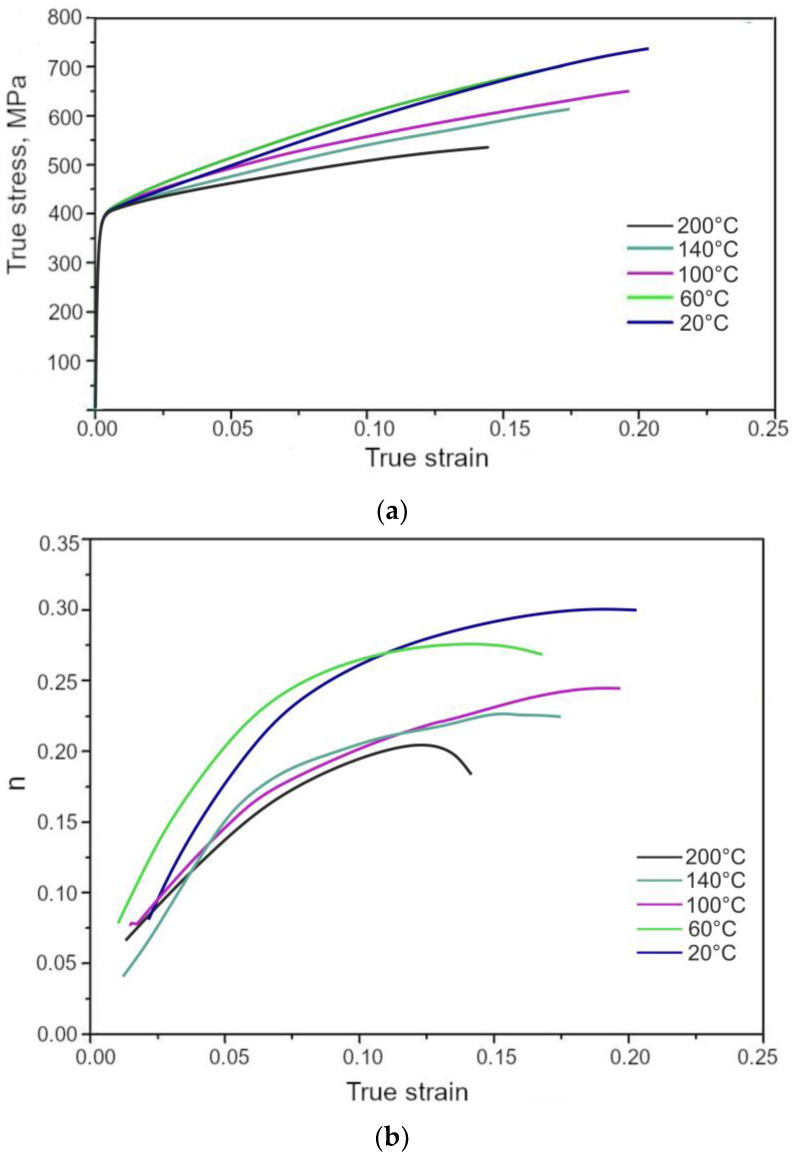
True stress vs. true strain (**a**) and work hardening exponent (*n*) changes (**b**) at different deformation temperatures.

The mechanical properties of the investigated steel are affected by the tendency of the γ phase to SIMT, which is substantially dependent on the deformation temperature. The true stress vs. true strain curves generated at different deformation temperatures are presented in Figure 11a. The real σ-ε data are valid in the uniform elongation range [41]. The mechanical properties as a function of the deformation temperature are summarized in Table 2.

**Figure 12:**
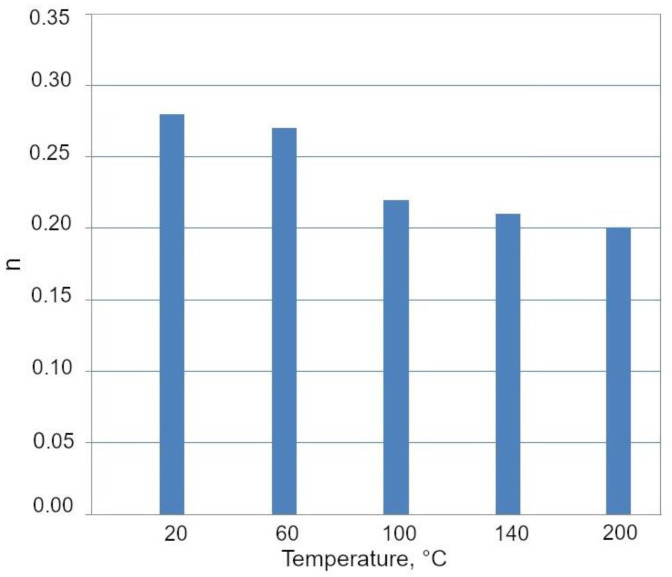
The highest *n* values at different deformation temperatures.

The deformation temperature has little effect on the yield stress (YS) in Figure 11a. Similar values of the YS were obtained independently on the deformation temperature. The ultimate tensile strength (UTS) decreased continuously with an increase in deformation temperature. A similar effect was reported by Jimenez et al. [22] in 0.22C-1.64Mn-1.51Al-0.05Si TRIP steel deformed between 100 and 150 °C. The highest UTS showed a specimen deformed at 20 °C (677 MPa). At this deformation temperature, the progressive transformation of RA into martensite during deformation provided a maximum *n* value (Figure 11b and Figure 12) at the relatively high true strain of 0.20. A gradual transformation of RA at 20 °C is also reflected in the highest value of uniform elongation (UEl) and total elongation (TEl), (23.5% and 26.3%, respectively). Moreover, the most beneficial YS/UTS ratio (0.65) and UTS × TEl product were noted for the specimen deformed at room temperature. Jimenez et al. [22] reported that the highest UEl was at a deformation temperature corresponding to the most intense TRIP effect. The amount of retained austenite transformed into martensite was gradually decreasing for the deformation temperatures above 20 °C (Figure 10) leading to the reduction in the TRIP effect. Therefore, a lower strain hardening ability (Figure 12) as well as reduced ductility (Table 2) were observed at elevated temperatures (60–200 °C). Results of studies presented by other authors [3,13,14] showed that satisfactory ductility of multiphase steels resulted both from the soft ferrite and the TRIP effect.

## 4. Conclusions 

A thermomechanically processed 0.24C-1.5Mn-0.87Si-0.4Al-Nb-Ti TRIP-aided alloy was tensile deformed at temperatures ranging from 20 to 200 °C. Relationships between the deformation temperature, morphological features of structural constituents, and mechanical properties were analyzed. The following conclusions were drawn:

- An increase in the deformation temperature resulted in the reduced SIMT due to the increasing stability of the γ phase. The percentage amount of RA transformed into martensite at 20 °C was 65%, whereas only 25% of the phase was subjected to the transformation at 200 °C;
- The TRIP effect played an important role in improving the mechanical behavior of the investigated steel. The SIMT operated in the whole temperature range but its intensity was much less at 140 and 200 °C. The interlath retained austenite maintained stability in this temperature range;
- The gradual SIMT of the retained austenite provided the beneficial balance of strength and ductility. The highest effectiveness of the TRIP effect was evidenced at 20 °C; thus, the best mechanical properties were noted at this temperature: the YS, UTS, TEl, and UEl values were: 437 MPa, 677 MPa, 26.3%, and 23.5%, respectively.

## Figures and Tables

**Table 1:** Chemistry of the alloy in wt.%.

C	Mn	Si	Al	P	S	Nb	Ti	N
0.24	1.50	0.87	0.40	0.010	0.004	0.034	0.023	0.0028

**Table 2:** Tensile test results of the investigated alloy at different deformation temperatures.

Deformation Temperature, °C	YS, MPa	UTS, MPa	TEl, %	UEl, %	YS/UTS	UTS × TEl, MPa%
20	437	677	26.3	23.5	0.65	17,805
60	431	644	23.9	18.2	0.67	15,391
100	428	583	24.3	21.0	0.73	14,166
140	426	563	23.3	19.1	0.76	13,117
200	411	532	16.9	15.3	0.77	8990
